# The course of Rotavirus A (RVA) infection in young racing pigeons during the racing season

**DOI:** 10.1186/s12917-024-04144-2

**Published:** 2024-07-09

**Authors:** Krzysztof Adamczyk, Aleksandra Ledwoń, Michał Czopowicz, Piotr Szeleszczuk

**Affiliations:** 1 Department of Pathology and Veterinary Diagnostics Warsaw, University of Life Sciences, Nowoursynowska 159C, Warsaw, 02-776 Poland; 2 Division of Veterinary Epidemiology and Economics, Institute of Veterinary Medicine, Warsaw University of Life Sciences, Nowoursynowska 159C, Warsaw, 02-776 Poland

**Keywords:** Young pigeon disease, Pigeon rotavirus, Pigeon, Rotavirus A, Pigeon races

## Abstract

**Background:**

Pigeon Rotavirus A (RVA) infection has been confirmed in pigeons in the last decade as a cause of Young Pigeon Disease (YPD). Although YPD has been known for many years to date, no studies have been conducted to track the spread of RVA infection in pigeons during the racing season. The presented research aims to determine the course of RVA infection during the flights of young racing pigeons in the summer season, in one of the districts in the Mazovian Voivodeship in Poland.

**Results:**

Faecal samples of pigeons collected from transport baskets in vehicles transporting pigeons to the starting point were tested. The quantitative RT-PCR (qRT-PCR) was used to detect the genetic material of RVA. Samples taken during 6 flights were analysed. The study showed a percentage increase in infections up to the fourth flight of pigeons, and then their decrease. With Cq values below 20, breeders did not participate in the next flight and/or reported disease in the flock. With positive Cq values of 20 to 30, clinical signs of disease were not reported. Of the 76 breeders participating in the races, at least one positive result was found in 46 (60.5%). Including the occurrence of the disease during the racing season was reported by 11 breeders (14.4%). The main clinical signs in sick pigeons were vomiting, diarrhea and stowed crop. The tested pigeons were not vaccinated against RVA.

**Conclusions:**

During training and racing of pigeons, it is not possible to avoid exposing them to pathogens, including RVA, regardless of whether pigeons from different breeders are placed in the same baskets or are in separate baskets. However, after four flights the number of new cases of the disease decreases which indicates the development of immunity. The qRT-PCR test is useful in the diagnosis and differentiation of clinical (Cq below 20) and subclinical RVA infections in racing pigeons.

## Background

Pigeon rotavirus A (RVA) has been diagnosed as a pathogenic agent for pigeons only recently [1–3], although young pigeon disease (YPD), of which RVA turned out to be a primary cause, has been known for many years [4, 5]. RVA infections have been diagnosed in both racing and fancy pigeons mainly in Europe [1, 6–8], Australia [2, 5, 9], the United States of America [10, 11], and Nigeria [12]. Young pigeons aged from 4 weeks to 6 months [2, 5] are susceptible to the disease. Clinical signs of YPD are mainly: regurgitation and vomiting, retention of contents in the crop (stowed crop), biliverdinuria and diarrhea. Mortality reaches 45% [2], and in ornamental pigeons even 75% [13]. Affected birds usually died within 12 to 24 h from onset of vomiting, with deaths continuing for about seven days [2]. Gross pathological lesions were: mildly enlarged, diffusely mottled or congested livers and sometimes enlarged, friable and mottled spleens and in some cases pale kidneys [2, 7]. For racing pigeon breeders, rotavirus is important mainly during the young pigeon racing season, when it significantly affects their performance, including the number of pigeons that do not return to the loft [4, 5]. Young racing pigeons become infected mainly during transport to the places where they are released for the race [2]. Transport to the starting point usually takes place in special trailers or semi-trailers of trucks with well-ventilated baskets made of generously perforated sheet metal or wire, tightly fitting to each other. The birds are placed in the transport cabin the day before the flight, usually on Saturday. The transportation occurs overnight, and the birds start their flight the next morning. The birds are given water during transport. Feeding is not conducted during the young pigeons flights due to the shorter distances they are transported. Therefore, the most likely route of infection of young pigeons during transport is water contaminated with faeces.

The aim of the study is to track the spread of YPD during training flights and races by transporting pigeons together to the start site.

## Materials and methods

### Samples and questionnaires

In the young pigeons racing season, from August to September, faecal samples were collected from the transport baskets after the pigeons left them during two training flights and five competition flights (races). The samples were tested for the presence of pigeon RVA genetic material except race five (7th flight). Samples from training flights on August 8 and 15 and races on August 11, 18, 25 and September 1, were analysed. Pigeons from individual breeders were usually placed in separate baskets. However, due to lack of space, pigeons from different lofts were combined in some baskets. Up to 20 pigeons were usually placed in one basket. Pigeons belonging to 76 breeders from the Mazovian Voivodship took part in the flights. The number of breeders participating in individual flights increased from 12 breeders in the first flight to 62 breeders in the sixth flight (Table 1). Before the flights, breeders were asked to fill in questionnaires regarding the flock health status: vaccination against RVA, presence of YPD before or during the flights, age of affected pigeons, and the presence of clinical signs typical of RVA infection, such as sudden deaths, diarrhea, vomiting, stowed crop, anorexia, weight loss [1, 2, 5] as well as atypical of this disease such as: polyuria, respiratory signs or nervous signs. Based on the aforementioned signs, a numerical score indicating that the presenting disease could be caused by RVA infection was developed (henceforward referred to as clinical score)—1 point was added for each typical clinical sign, while 1 point was subtracted for each atypical sign. Therefore, the score ranged from -3 to 6. In addition, mortality and morbidity rates, and information about the date when clinical signs first occurred were requested from the owners. In total, 52 breeders returned a completed questionnaire at least once. If the breeder observed clinical signs in the pigeons in the breeding, an additional sample was collected from the loft.

**Table 1:** A summary of results of qRT-PCR for the presence of rotavirus A (RVA) in faecal samples collected from baskets of individual lofts in subsequent flights, reported clinical signs, and the lack of participation of pigeons in the next flight after RVA detection

Flight number	No. of lofts taking part in the flight	Lofts with positive RVA qRT-PCR results		No. of Cq results below 20	Reported YPD signs during or after flight	No. of breeder participating in the next flight after RVA detection
		No	Proportion (CI 95%) [%]			
1	12	3	25 (9 – 53)	1/3	1	1
2	35	6	17 (8 – 33)	1/6	0	2
3	31	8	26 (14 – 43)	3/8	3	3
4	36	15	42 (27 – 58)	4/15	6	6
5	52	16	31 (20 – 44)	1/16	1	2
6	62	7	11 (6 – 22)	0/7	0	3

*CI 95%* 95% confidence interval

### RNA extraction and quantitative RT-PCR

From one transport basket, up to 25 g of faeces were collected into 50 ml centrifuge tubes (Sarstedt, Germany). A volume of 1 × PBS Buffer (Eurx, Poland) equal to twice the sample volume (usually about 50 ml) was then added. After vortexing for approximately 1 min, the samples were centrifuged for 15 min at 3779 RCF. After centrifugation, 100 µl of the supernatant was collected for RNA isolation using the Total RNA mini kit (A&A Biotechnology, Poland) according to the manufacturer's instruction.

Detection of pigeon RVA was performed by a previously published VP6-specific qRT-PCR assay [2] modified by Rubbenstroth et al. [3] with AgPath-ID One-Step RT-PCR (Thermo Fisher Scientific, USA). VP6-specific qRT-PCR was performed using CoRVA_VP6_868 + and CoRVA_VP6_943-primers and probe 3-CorVA_VP6_898_P [3]. Threshold cycle (Cq) scores below 30 were considered positive, scores from 31 to 35 were considered weakly positive [3] and were not taken into account.

### Statistical analysis

Numerical variables were presented as the median and range (or interquartile range in figures), and compared between unpaired groups using the Mann–Whitney U test. Correlation between numerical variables was determined using Spearman’s rank correlation coefficient (R_s_). Categorical variables were expressed as the count and percentage in a group. Proportions were compared between unpaired groups using the maximum likelihood G test or Fisher exact test if the expected count in the contingency table was < 5. The χ2 test for trends was used for comparing proportions between subsequent time points. The 95% confidence intervals (CI 95%) for proportions were calculated using Wilson score method. All tests were two-tailed and a significance level (α) was set at 0.05. Statistical analysis was performed in TIBCO Statistica 13.3 (TIBCO Software Inc., Palo Alto, CA, USA).

## Results

### qRT-PCR test results

**Fig. 1:**
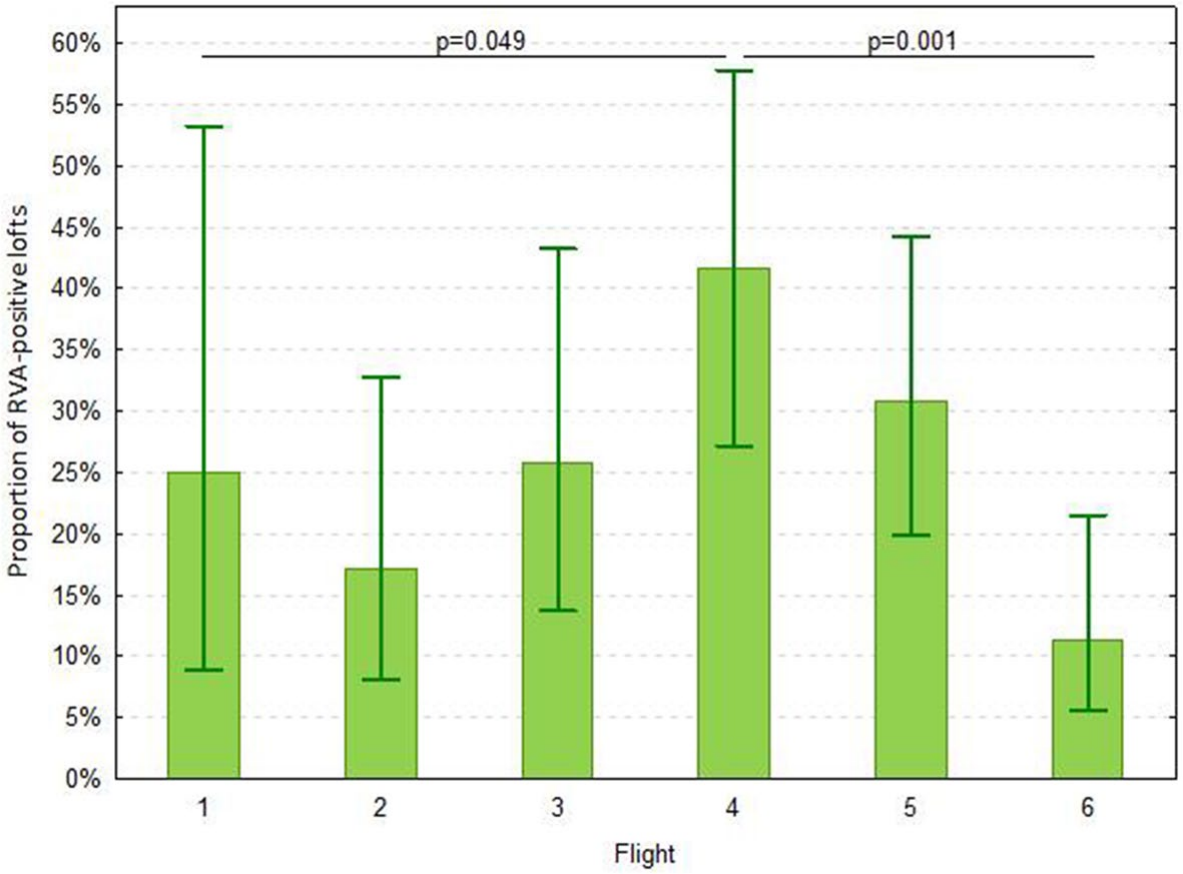
Proportion of positive results of qRT-PCR results for the presence of rotavirus A (RVA) in faecal samples collected from baskets of individual breeders in subsequent flights. Whiskers indicate the 95% confidence interval

At least one positive result of qRT-PCR for RVA was found in 46 of 76 lofts owned by different breeders participating in the races (61%, CI 95%: 49% – 71%). The proportion of positive results significantly increased until the fourth flight (*p* = 0.049) (Table 1, Fig. 1), and then significantly decreased until the sixth flight (*p* = 0.001). The decrease was observed even though the number of shared baskets was even higher than in previous flights (Table 2). Eleven breeders reported on the occurrence of YPD during the races, while 17 breeders with a positive RVA result in a given race did not participate in the next race. In pigeons from 12 breeders, an earlier negative result was followed by a positive RVA result. Depending on the flight date, the period between these results was 3–7 days. Pigeons from lofts with post-flight disease cases showed clinical signs 1–7 days after the flight in which the basket sample was positive. Additional loft samples were collected from the 10 lofts where the breeders reported clinical signs. In four cases, the qRT-PCR yielded a Cq below 20, in four cases between 20 and 30, and in two cases the result was negative. In one case the loft sample was negative, but the transport basket sample was positive. There was no significant difference in the proportion of RVA-positive results for baskets populated with pigeons of a single breeders and baskets populated with pigeons of several different breeders (Table 2).

**Table 2:** A summary of results of qRT-PCR for the presence of rotavirus A (RVA) in faecal samples collected from baskets with pigeons belonging to one breeder (single-breeder baskets) and baskets with pigeons from various breeders (shared baskets) in subsequent flights

Flight	Total number of baskets	Number of		RVA-positive (CI 95%)				
		single-breeder baskets	shared baskets	single-breeder baskets		shared baskets		*p*-value
				n	% (CI 95%)	n	% (CI 95%)	
1	36	36	0	4	11 (4 – 25)	-	-	-
2	124	122	2	10	8 (5 – 14)	0	0 (0 – 66)	0.999
3	58	37	21	11	30 (17 – 46)	3	14 (5 – 35)	0.174
4	96	93	3	30	32 (24 – 42)	1	33 (6 – 79)	0.999
5	63	48	15	14	29 (18 – 43)	5	33 (15 – 58)	0.760
6	85	67	18	7	10 (5 – 20)	1	6 (1 – 26)	0.999

*CI 95%* 95% confidence interval

### Results of the questionnaire analysis

**Fig. 2:**
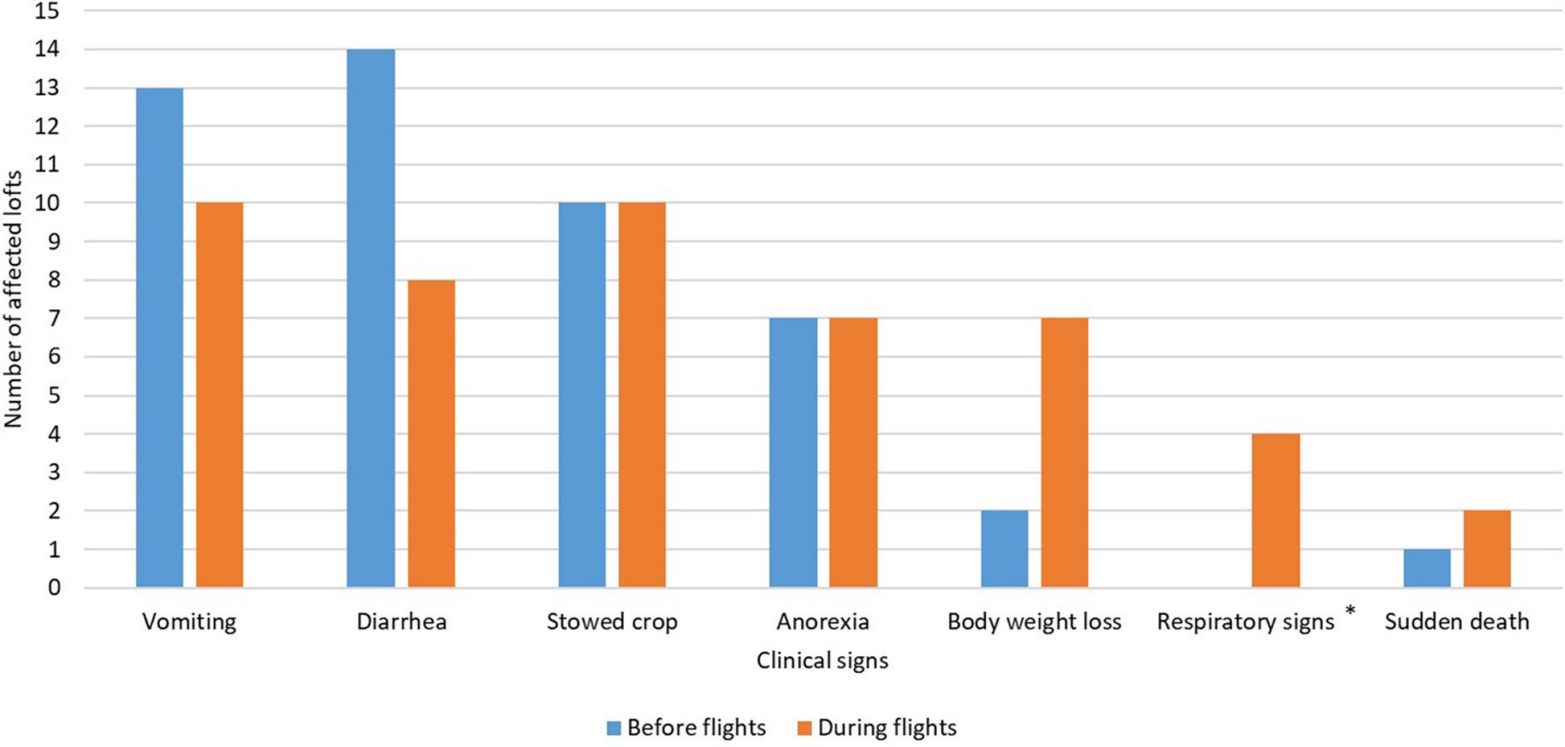
Clinical signs observed in loft affected by Young Pigeon Disease (YPD), before and during flights. Asterisk indicates the sign atypical of YPD

**Fig. 3:**
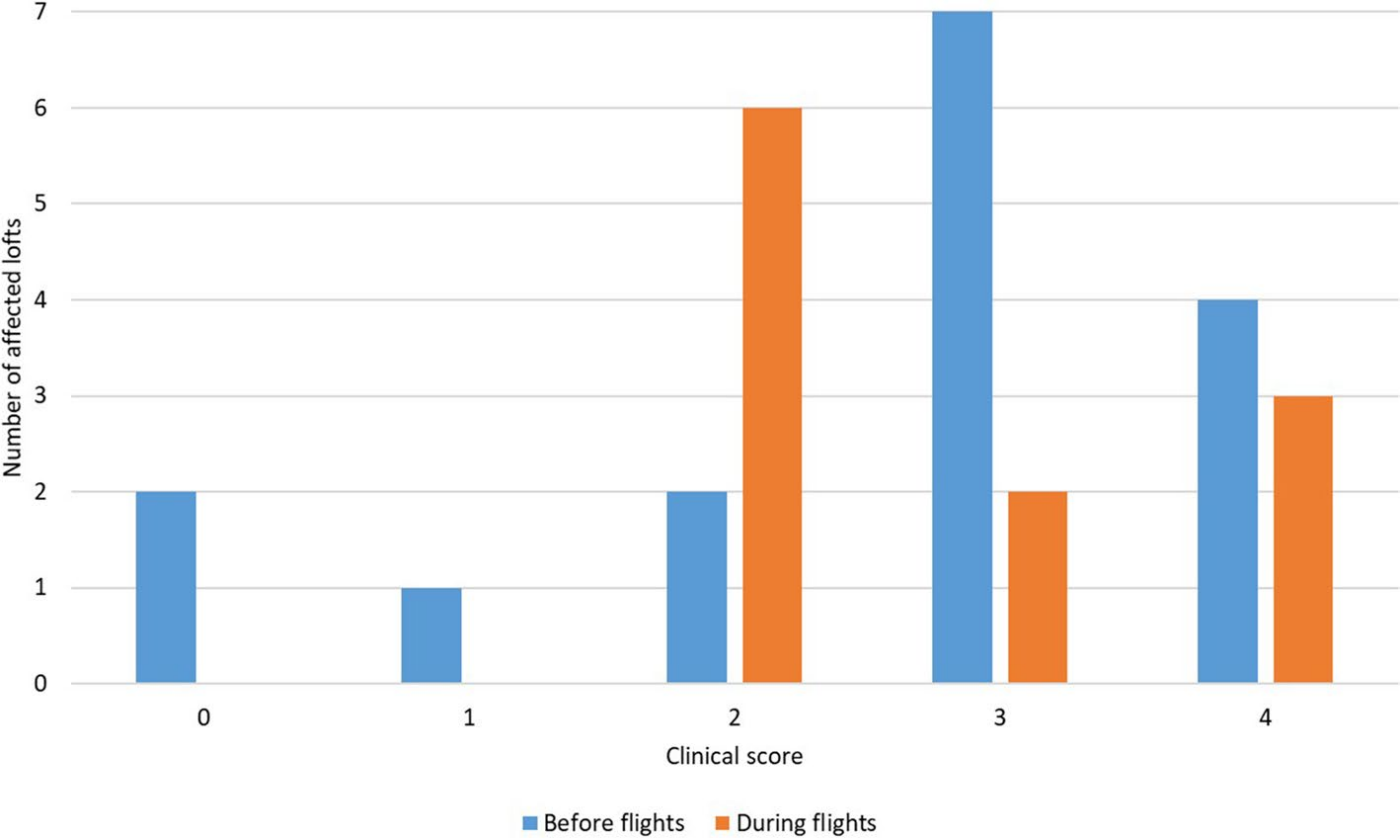
Clinical score observed in lofts affected by Young Pigeon Disease, before and during flights

Of 52 breeders who returned questionnaires, 27 reported illness before or during the flights (52%, CI 95%: 39% – 65%). One breeder observed YPD clinical signs twice, before and during the flights. The most commonly observed clinical signs were vomiting, diarrhea, and stowed crop (Fig. 2). The scores of clinical signs varied depending on the time of onset of the disease. In pigeons ill before the flights, the score was usually 3/6, while in pigeons ill during the flights it was 2/6 (Fig. 3). The tested pigeons were not vaccinated against RVA.

### Comparison of qRT-PCR results with questionnaire data

Cq values in the qRT-PCR test were significantly lower in pigeons with a clinical score > 0 (median 19, range 13 – 30) than in pigeons with no clinical signs (median 28, range 16 – 30; *p* = 0.002). However, clinical score was not correlated with qRT-PCR-Cq (*R*_s_ = -0.02; *p* = 0.949). In sixteen lofts (21%) in which pigeons took part in flights, breeders had previously reported a disease similar to YPD. In four cases, the qRT-PCR results of these pigeons were negative during the whole study period. In five cases, pigeons were withdrawn from the subsequent flight after the positive qRT-PCR result. In 7 cases, pigeons took part in the next flight despite the positive qRT-PCR result. Only one breeder in this group reported the flock becoming ill during flight. Of the 31 breeders who did not report a previous case and were found to be positive for RVA, 15 did not participate in the subsequent flight after testing positive, while 16 did. The incidence of YPD during the season was significantly higher in these flocks in which YPD before the season did not occur (32%; CI 95%: 19% – 50%; 10 / 31) compared to the flocks in which YPD occurred before the season (7%; CI 95%: 1% – 30%; 1 / 15; *p* = 0.039).

## Discussion

A number of factors influence the development of RVA infection in pigeons during the racing season. The most important are the contact with infected birds during transport, the introduction of infected birds into the loft [2, 5], and the stress induced by the contact with other unknown pigeons [4, 5, 14]. As a consequence of YPD outbreaks, racing pigeon fanciers often report sudden loss of racing performance and increased loss of animals during flights [4, 5]. In our study, the faeces of pigeons from 61% of lofts were positive, while in the study by Harzer et al. [13], RVA test was positive in swabs from only 10% of fancy pigeon flocks. These differences are likely due to the decreased contact of fancy pigeons with pigeons from other lofts outside the exhibition season, as the introduction of the disease takes place largely through newly acquired birds to the loft. In our case, only 14% of breeders reported pigeon disease during flights and 21% before flights, while in previous studies, as many as three fourths of breeders reported pigeon disease after the exhibition [13]. This discrepancy may result from a longer period of exposure of pigeons to infection at exhibitions, which may last up to several days. In our study, the most objective method of infection monitoring was the examination of faecal samples using the molecular method and the observation whether the pigeon breeder with a positive result took part in the next flight or reported sick birds. The questionnaires were not always filled in by breeders. Also, the absence in the next flight did not always have to be associated with the disease of the birds. Nevertheless, information on 27 YPD cases was obtained.

The present study determined whether the birds participating in the flights were already infected with the virus and, if the breeder reported it, at when the disease occurred. In some cases, when pigeons participated multiple times, positive results could be found after previously negative results. In cases reported by other authors, symptoms of YPD were observed six to seven days following the race [1] and 1 to 4 days after visiting a show [13]. Disease in the flocks of pigeons we studied usually occurred 1–7 days after the flight. According to the information obtained from breeders, the morbidity and mortality in lofts with confirmed RVA infection during flights was 43%-100% and 3%-50%, respectively. In fancy pigeon flocks studied by other authors, the morbidity ranged from 8 to 100%, while the mortality rate ranged from 0 to 75% [13], also the same authors found positive RVA RT-PCR results in pigeons of a breeder who did not observe symptoms of the disease in the loft [13]. In the cases we studied, some breeders were also positive despite the absence of clinical signs, but the reliable indicator was the Cq values, which, reaching values below 20, almost guaranteed the occurrence of clinical signs. The clinical signs most commonly reported by breeders are vomiting, diarrhea, stowed crop, anorexia, and weight loss, respectively. Vomiting, regurgitation and diarrhea are the symptoms described by some authors [5, 9, 10,], while in other studies [1, 3], anorexia was the most frequently reported symptom. Morbidity and mortality in lofts participating in the described flights ranged from 3 to 100% and from 0 to 50%, respectively. In studies conducted by other authors, it was: ≤ 100% and 0–45% [2, 3, 8, 9]. In one of the cases described by Harzer et al. mortality was even 75%, however, it is possible that such high mortality in this flock could be caused by co-infections [13].

## Conclusions

During training and flights of pigeons, it is impossible to avoid their exposure to pathogens, including rotavirus A. Our research shows that after just four flights, the percentage of new cases of the disease decreases, which indicates the development of immunity. Testing for the presence of RVA RNA in pigeon faeces using the qRT-PCR test is useful in the diagnosis and differentiation of clinical (Cq below 20) and subclinical RVA infections in racing pigeons. Moreover, the course of RVA infection in racing pigeons is similar, regardless of whether pigeons from different breeders are placed in the same baskets or in separate baskets during the same flight.

## Data Availability

No datasets were generated or analysed during the current study.
